# Rapid development of cross-clade neutralizing antibody responses after clade B gp120/gp140 protein priming and clade c gp140 protein boosting

**DOI:** 10.1186/1742-4690-9-S2-P137

**Published:** 2012-09-13

**Authors:** P Spearman, G Tomaras, D Montefiori, Y Huang, H Ahmed, M Elizaga, J Hural, J McElrath, L Ouedraogo, M Pensiero, C Butler, S Kalams, ET Overton, S Barnett, N Group

**Affiliations:** 1 Emory University, Atlanta, GA, USA; 2 Department of Surgery, Duke Human Vaccine Institute, Durham, NC, USA; 3 Fred Hutchinson Cancer Research Center, Seattle, WA, USA; 4 Division of AIDS, NIAID, NIH, Bethesda, MD, USA; 5 Vanderbilt University School of Medicine, Nashville, TN, USA; 6 University of Alabama at Birmingham, Birmingham, AL, USA; 7 Novartis Vaccines and Diagnostics, Cambridge, MA, USA

## Background

Immunization with heterologous Env protein immunogens following an immunologic rest period has the potential to generate cross-clade neutralizing antibody responses. We identified individuals who had received a clade B Env protein with MF59 4-17 years earlier, most in combination with a DNA or ALVAC prime, and administered a clade C protein boost in an open label phase 1 trial.

## Methods

Sixteen previously primed volunteers and 20 naïve volunteers each received 2 doses of a clade C TV1 trimeric Env protein with MF59 given 6 months apart. HIV-1 specific CD4+ and CD8+ T cell responses were measured by an intracellular cytokine staining (ICS) assay. Antibody responses were measured with a Luminex binding antibody assay and a neutralizing antibody assay in TZM-bl Cells.

## Results

Despite the long interval, 31% of primed participants demonstrated CD4+ T cell responses to Env at baseline, which increased to 75% after a single protein boost. IgG and IgA responses to TV1 trimeric Env were present in 64% (IgG) and 7% (IgA) of primed participants at baseline, and rose to 93% and 85%, respectively, after one dose of protein. 71% of primed participants demonstrated neutralizing antibodies against Tier 1 clade B isolate MN at baseline. After a single booster dose of protein, 100% of the primed participants neutralized MN and 93% showed neutralizing activity against a clade C isolate, MW965.26. Unprimed participants did not demonstrate CD4+ responses or antibody responses to Env until after the second dose, which elicited IgG and IgA responses to TV1 trimeric Env in 88% and 50%, respectively. Neutralizing antibody developed to MN in 38% and to MW965.26 in 88% of the unprimed participants.

## Conclusion

These results demonstrate the durability of vaccine-elicited HIV-1 specific antibody responses and support current efforts to enhance the breadth and magnitude of neutralizing antibodies through heterologous protein prime-boost regimens.

